# Editorial: Hot topic: anti-inflammatory drug discovery

**DOI:** 10.3389/fchem.2024.1512607

**Published:** 2024-11-06

**Authors:** Abdul Ajees Abdul Salam, Tejas M. Dhameliya, Ahmed Mahal, Sajad Moradi, Paolo Coghi

**Affiliations:** ^1^ Department of Atomic and Molecular Physics, Manipal Academy of Higher Education, Manipal, Karnataka, India; ^2^ Department of Pharmaceutical Chemistry, Institute of Pharmacy, Nirma University Ahmedabad, Ahmedabad, Gujarat, India; ^3^ Department of Medical Biochemical Analysis, College of Health Technology, Cihan University-Erbil, Erbil, Iraq; ^4^ Nano Drug Delivery Research Center, Health Technology Institute, Kermanshah University of Medical Sciences, Kermanshah, Iran; ^5^ School of Pharmacy, Faculty Medical Division, Macau University of Science and Technology, Macau, China

**Keywords:** inflammation, *Euphorbia neriifolia L*, *Zanthoxylum simulans hance*, *Boerhavia diffusa L*, *Chamomilla recutita (L) Rausch*

Inflammation is a common pathological process underlying a range of chronic conditions, from cancer to autoimmune diseases, and the search for natural anti-inflammatory agents continues to reveal promising leads. The latest research on bioactive compounds derived from medicinal plants such as *Euphorbia neriifolia L*. (Chang et al.), *Zanthoxylum simulans* (Tian et al.)*, Boerhavia diffusa L*. (Das et al.) and *Chamomilla recutita L.* (Lairikyengbam et al.) brings exciting new insights into the molecular mechanisms of inflammation control. These discoveries underline the potential of phytochemicals as therapeutic agents with fewer side effects compared to conventional drugs.

One of the most intriguing findings comes from the study of *E. neriifolia L*., (Chang et al.), a plant long valued in traditional medicine across India and Southeast Asia. In a recent study, eight triterpenes were isolated from the ethanolic extract of the plant’s stem, six of which were new compounds. The euphane-type triterpenes exhibited selective inhibition of interleukin-6 (IL-6) production in LPS-induced macrophage cells, highlighting their role as targeted anti-inflammatory agents. The study also identified tirucallane-type triterpene (neritriterpenol I) as a potent inhibitor of both IL-6 and tumor necrosis factor-alpha (TNF-α). This selective anti-inflammatory action opens up possibilities for the development of compounds that modulate specific inflammatory pathways, potentially reducing side effects associated with broader immune suppression.

Similarly, research on quaternary alkaloids from *Z. simulans* (Tian et al.), has identified two potent dual inhibitors of cyclooxygenase-2 (COX-2) and 5-lipoxygenase (5-LOX)—key enzymes in inflammation and cancer metastasis. Among these, chelerythrine showed significant time- and dose-dependent inhibition of AGS gastric cancer cell proliferation. Its efficacy is linked to the modulation of hormone signaling pathways, particularly those involving estrogen, thyroid, and oxytocin receptors. This integrative approach not only enhances our understanding of chelerythrine’s anti-cancer potential but also emphasizes the importance of a balanced hormone-signaling response when targeting inflammation in cancer therapy.

The ethnopharmacological relevance of *Boerhavia diffusa L.,* (Das et al.), commonly known as Punarnava, highlights another medicinal plant with broad anti-inflammatory, antioxidant, and anticancer properties. This herb has been used for centuries in Ayurveda for its rejuvenating properties, but modern pharmacological studies have unveiled a host of bioactive compounds responsible for its therapeutic effects. Phenolics, flavonoids, and alkaloids punarnavoside and rotenoid boeravinones are now recognized for their potential to combat inflammation and oxidative stress. Further research using molecular docking studies has validated these phytocomponents as viable candidates for drug development, adding scientific credence to their traditional use.

Lastly, the anti-inflammatory properties of chamomile (*C. recutita L*.) (Lairikyengbam et al.) have long been recognized, but recent investigations into its effects on T cells offer a deeper understanding of its role in treating chronic inflammatory diseases. By inhibiting T cell activation and reducing cytotoxic granzyme B production, chamomile extracts and pure compounds like apigenin show potential for treating inflammatory skin diseases and chronic wounds. The study also underscores the importance of extraction methods, as different preparations resulted in varying phytochemical compositions and, consequently, different impacts on T cell activity. This points to the necessity of standardizing extract preparations for therapeutic consistency.

These studies collectively reinforce the vast therapeutic potential of medicinal plants in combating inflammation. By unveiling the molecular pathways modulated by phytochemicals, researchers are bringing traditional medicine into the realm of modern therapeutics. However, the path from bench to bedside requires further exploration, especially concerning bioavailability, efficacy in human trials, and the standardization of natural extracts. As we continue to uncover nature’s pharmacy, it becomes increasingly clear that these plants hold promise for developing safer, more targeted treatments for inflammation-driven diseases. This wealth of phytochemical diversity, combined with advances in bioinformatics and molecular docking, signals a bright future for natural product-based drug discovery in the fight against chronic inflammation and related pathologies.

